#  Concurrent/sequential versus sequential immune checkpoint inhibition in inoperable large stage III non-small cell lung cancer patients treated with chemoradiotherapy: a prospective observational study

**DOI:** 10.1007/s00432-023-04654-w

**Published:** 2023-03-20

**Authors:** Lukas Käsmann, Chukwuka Eze, Julian Taugner, Alexander Nieto, Kerstin Hofstetter, Sophie Kröninger, Julian Guggenberger, Saskia Kenndoff, Benedikt Flörsch, Amanda Tufman, Niels Reinmuth, Thomas Duell, Claus Belka, Farkhad Manapov

**Affiliations:** 1 Department of Radiation Oncology, University Hospital, University of Munich (LMU), Munich, Germany; 2 Comprehensive Pneumology Center Munich (CPC-M), Member of the German Center for Lung Research (DZL), Munich, Germany; 3 German Cancer Consortium (DKTK), Partner Site Munich, Munich, Germany; 4 Division of Respiratory Medicine and Thoracic Oncology, Department of Internal Medicine V, Thoracic Oncology Center Munich, University of Munich (LMU), Munich, Germany; 5 Asklepios Lung Clinic, Gauting, Munich, Germany

**Keywords:** NSCLC, Durvalumab, Nivolumab, Chemoradiation, Sequence

## Abstract

**Purpose/aim:**

The international standard for patients with large inoperable stage III NSCLC is durvalumab consolidation after concurrent chemoradiotherapy (CRT). In this single centre observational study based on individual data, we prospectively evaluated the role of concurrent/sequential versus sequential immune checkpoint inhibition (ICI).

**Methods and patients:**

In total, 39 stage III NSCLC patients were prospectively enrolled, 11 (28%) patients were treated with simultaneous and consolidation therapy with PD-1 inhibition (nivolumab) (SIM-cohort) and 28 (72%) patients received PD-L1 inhibition (durvalumab) as consolidation treatment up to 12 months after the end of CRT (SEQ-cohort).

**Results:**

For the entire cohort, median progression-free survival (PFS) was 26.3 months and median survival (OS), locoregional recurrence-free survival and distant metastasis-free survival were not reached. For the SIM-cohort, median OS was not reached and PFS was 22.8 months, respectively. In the SEQ-cohort, neither median PFS nor OS were reached. After propensity score matching, PFS at 12/24 months were 82/44% in the SIM-cohort and 57/57% in the SEQ-cohort (*p* = 0.714), respectively. In the SIM-cohort, 36.4/18.2% of patients showed grade II/III pneumonitis; in the SEQ-cohort 18.2/13.6% after PSM (*p* = 0.258, *p* = 0.55).

**Conclusion:**

Both concurrent/sequential and sequential ICI show a favorable side effect profile and promising survival in treated patients with inoperable large stage III NSCLC. Concurrent ICI showed a numerical non-significant improvement regarding 6- and 12-months PFS and distant control compared to sequential approach in this small study. However, concurrent ICI to CRT was associated with a non-significant moderate increase in grade II/III pneumonitis.

**Supplementary Information:**

The online version contains supplementary material available at 10.1007/s00432-023-04654-w.

## Introduction

Locally advanced non-small-cell lung carcinoma (NSCLC) is characterized by a highly heterogeneous group of patients regarding tumor localization and nodal involvement (Unterrainer et al. 2021; Kaesmann et al. 2019). As a result, patients prognosis ranges from 13 to 30 months in relation to median overall survival (OS) and 5-year survival rates from 15 to 35% in real-life settings, respectively (Yusuf et al. 2020; Taugner et al. 2021; Moore et al. 2019). In the last decade, normofractionated thoracic radiotherapy with a cumulative dose of at least 60 Gy and a concurrent platinum-based chemotherapy represents the optimal treatment in stage III NSCLC based on the results of RTOG 73-01, RTOG 9410, CALGB 8433 and RTOG 0617 (Perez et al. 1987; Dillman et al. 1996; Curran et al. 2011; Bradley et al. 2020). A new treatment paradigm has been established by the results of the pivotal PACIFIC trial revealing an improvement regarding OS and progression-free survival (PFS) due to a maintenance treatment of Programmed death-ligand 1 (PD-L1) inhibitor durvalumab after concurrent chemoradiation (CRT). Administering durvalumab after CRT resulted in a notable PFS benefit in all subgroups leading to further implementation of this multimodal treatment approach worldwide. Importantly, the European Medicines Agency (EMA) given the approval of durvalumab only in PD-L1 positive tumors (≥ 1%)] based on a post-hoc analysis (European Medicines Agency 2018).

Application of immune checkpoint inhibition (ICI) such as anti–programmed cell death-protein 1 (PD-1) or PD-L1 with radiotherapy may further improve local and distant tumor control in preclinical models based on a synergistic antitumor effect (Formenti and Demaria 2009; Vanpouille-Box et al. 2017; Alsaab et al. 2017). In fact, Dovedi et al. found that not sequential, but concurrent application of anti-PD-1/PD-L1 to radiotherapy results in durable tumor control (Dovedi et al. 2014). In addition, PD-L1 expression on tumor cells could be induced by irradiation based on several preclinical studies (Dovedi et al. 2014; Chen et al. 2016; Azad et al. 2017).

The first phase II trial regarding the concurrent administration of PD-1 inhibition was the NICOLAS trial combining nivolumab with concurrent normofractionated CRT followed by 12 months of nivolumab consolidation treatment (Peters et al. 2021).

In this first prospective study, we evaluated the role of simultaneous and consolidation therapy versus consolidation treatment alone with ICI in patients with large inoperable stage III NSCLC treated with CRT based on an observational study.

## Patients and methods

### Study population

**Fig. 1:**
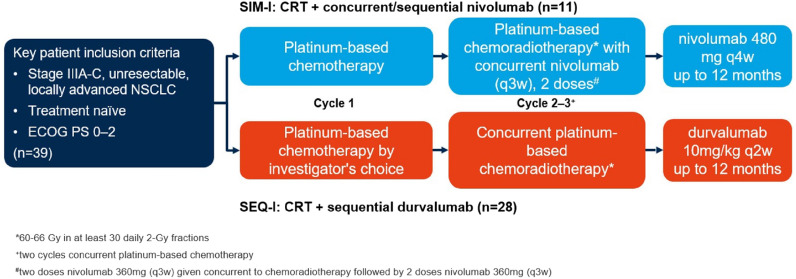
Study design of the prospective comparative study investigating concurrent/sequential versus sequential checkpoint inhibition in patients treated with chemoradiation in stage IIIA-C NSCLC

Between 10/1/2016 and 12/31/2020, 73 consecutive patients receiving platinum-based CRT for stage III A/B/C NSCLC (Union for International Cancer Control (UICC) 8th edition) were prospectively enrolled (see Supplementary Fig. 1). Hence, 39 (53%) patients received definitive CRT with either concurrent/sequential PD-1 inhibition (nivolumab) or consolidation treatment with PD-L1 inhibition (durvalumab) up to 12 months (see Fig. 1).

All patients were included and agreed to the use of their data for research purposes. In addition, the ethics committee of the Ludwig-Maximilians-University granted ethics approval for this study (17–230).

Every patient was discussed in a multidisciplinary tumor board and were considered inoperable based on the decision of experienced thoracic surgeons, medical oncologists/pulmonologists and radiation oncologists. Additional features such as general condition, comorbidities and tobacco consumption were assessed and critically considered. Exclusion criteria were a poor performance status (Eastern Co-operative Oncology Group performance status (ECOG PS) > 2), restricted lung function (DLCO < 40%, FEV1 < 1 l or on long-term oxygen therapy) and a planned non-curative RT (insufficient dose < 60 Gy). In the diagnostic and pre-treatment work-up, positron emission tomography (PET)-CT (*n* = 38) or contrast-enhanced computed tomography (CT) (*n* = 1) was performed. The majority of patients (84%, *n* = 33) received cranial contrast-enhanced magnetic resonance imaging (MRI) before CRT. In case of contradictions of MRI, patients received a contrast-enhanced head CT (*n* = 6).

### Immune checkpoint inhibition

Patients treated in the concurrent/sequential cohort were treated with an induction cycle of platinum-based chemotherapy before CRT (see Fig. 1). Two cycles of current platinum-based chemotherapy together with 360 mg nivolumab were administered during RT followed by 480 mg nivolumab as maintenance treatment every 4 weeks for up to 12 months until progressive disease or intolerable toxicity assessed with Common Toxicity Criteria for Adverse Events (CTCAE) version 5.

In the sequential cohort, patients received an induction chemotherapy by investigators choice (21,4%, *n* = 6, maximum of 2 cycles) and durvalumab every 2 weeks with a dose of 10 mg/kg up to one year (24 cycles) after the end of CRT and discontinued only in case of progression or unacceptable toxicity (CTCAE v5).

### Chemoradiation

Radiotherapy planning was performed incorporating features from PET-CT or contrast-enhanced diagnostic CT. Gross tumor volume (GTV), clinical target volume (CTV) and the planning target volume (PTV) were contoured using standing orders based on the latest published ESTRO ACROP guideline and PACIFIC trial protocol (Nestle et al. 2018; Antonia et al. 2018). In case of induction chemotherapy, the residual primary tumor volume was based on the planning PET-CT. However, initially involved lymph-node levels were covered in the PTV.

Conventionally fractionated radiotherapy with a median total dose ranging between 60 and 66 Gy was given to the primary tumor and the involved lymph node levels. Radiation delivery was performed in all patients using a linear accelerator (LINAC) with volumetric modulated arc therapy (VMAT). Cone-beam CT (CBCT) was routinely performed to assess inter-fraction motion.

### Patient follow-up

Follow-up consisted of clinical examinations, blood sample, lung function testing, and imaging (CT/PET-CT scans) every three months for up to 24 months after CRT and twice every year after for up to five years, based on our follow-up protocol. Routinely performed contrast-enhanced brain MRIs or bronchoscopy were not part of the follow-up protocol and conduced only if clinically indicated.

Index date of median follow-up, PFS, Infield and outfield recurrence free survival (IFRFS and OFRFS), distant metastasis free survival (DMFS), brain metastasis free survival (BMFS) and locoregional recurrence-free survival (LRFS) was the last date of CRT. Local recurrence pattern was determined as in-field (IFR) vs. out-of-field recurrence (OFR) based on the 50 Gy isodose line in the involved lung, respectively.

### Statistical analysis

Statistics were carried out using IBM SPSS version 26 (IBM, Armonk, NY, USA). Kaplan–Meier method and Cox regression analysis were used to compare both treatment groups. Thereafter, propensity Score Matching (PSM) using age, gender, performance status using ECOG PS, T category, PTV and histology was performed using the R plug-in for IBM SPSS 26 (Ho et al. 2007; Huang et al. 2015; Chao et al. 2017; Hansen 2004). A *p* value < 0.05 were considered significant.

## Results

In Table 1, all patient and tumor characteristics of the entire cohort, concurrent/sequential PD-1 inhibition (nivolumab subgroup) and sequential PD-L1 inhibition (durvalumab subgroup) are shown.

**Table 1:** Patient characteristics of the entire cohort, nivolumab and durvalumab subgroup

Parameter	Entire cohort *N* (%)	Nivolumab cohort	Durvalumab cohort	*p* value
Total	39	11	28	N/A
Age, years				
Median (range)	64 (44–78)	59 (45–77)	68 (44–78)	0.078
Gender				
Male	28 (72)	8 (73)	20 (71)	
Female	11 (28)	3 (27)	8 (29)	0.935
Eastern co-operative oncology group performance status (ECOG PS)				
0	22 (56)	7 (64)	15 (54	
1	15 (38)	4 (36)	11 (39)	
2	2 (5)	0 (0)	2 (7)	0.623
Tobacco consumption				
< 40 packyears	20 (51)	8 (73)	12 (43)	
≥ 40 packyears	19 (49)	3 (27)	16 (57)	0.093
Continued smoking				
No	27 (69)	8 (73)	19 (68)	
Yes	12 (31)	3 (27)	9 (32)	0.767
T-stage				
1	3 (8)	2 (18)	1 (4)	
2	5 (13)	2 (18)	3 (11)	
3	11 (28)	1 (9)	10 (36)	
4	20 (51)	6 (55)	14 (50)	0.208
N-stage				
0	7 (18)	0 (0)	7 (25)	
1	3 (8)	0 (0)	3 (11)	
2	14 (36)	2 (18)	12 (43)	
3	15 (39)	9 (82)	6 (21)	< 0.001
UICC 8				
IIIA	12 (31)	0 (0)	12 (43)	
IIIB	19 (49)	6 (55)	13 (46)	
IIIC	8 (21)	5 (46)	3 (11)	0.007
Histology				
Squamous cell carcinoma (SCC)	16 (41)	4 (36)	12 (43)	
Adenocarcinoma (ADC)	21 (54)	7 (64)	14 (50)	
Large cell carcinoma (LCC)	2 (5)	0 (0)	2 (7)	0.424
Concurrent platinum-based chemotherapy				
Yes	38 (97)	11 (100)	27 (96)	
No	1 (3)	0 (0)	1 (4)	0.525
Immune checkpoint inhibition				
Nivolumab	11 (28)	11 (100)	0 (0%)	
Durvalumab	28 (72)	0 (0)	28 (100)	N/A
Planning target volume (PTV)				
< 700 ccm	23 (59)	7 (64)	16 (57)	
≥ 700 ccm	16 (41)	4 (36)	12 (43)	0.711
< 900 ccm	32 (82)	10 (91)	22 (79)	
≥ 900 ccm	7 (18)	1 (9)	6 (21)	0.366
V20,%				
Mean, SD	24.1 (4.5)	26.0 (4.9)	23.4 (4.2)	0.083
MLD, Gy				
Mean, SD	13.9 (2.3)	14.8 (2.4)	13.5 (2.2)	0.048
PD-L1 status				
≥ 50%	15 (38)	2 (18)	13 (33)	
1–49%	15 (38)	1 (9)	14 (50)	
0%	2 (5)	2 (18)	0 (0)	
Unknown	7 (18)	6 (55)	1 (4)	< 0.001

The entire cohort consisted of 39 patients treated with CRT and ICI for inoperable stage III NSCLC.

Median age was 64 with 11 (28%) patients older than 70 years. Eleven (28%) were female and 28 (72%) male. On pre-treatment staging, 3 (8), 5 (11), 11 (28) and 20 (51) were suffering from T1, T2, T3, and T4 disease and 7 (18%) N0, 3 (8%) N1, 14 (36%) N2, and 15 (39%) N3 disease, respectively.

There were twelve (31%) patients with UICC stage IIIA, 19 (49%) patients IIIB and 8 (21%) patients IIIC. The median PTV was 676.0 ccm (range: 205–1235). The histo-pathalogical assessment shows that 16 (41%) patients had squamous-cell-carcinoma (SCC), 21 (54%) had adenocarcinoma (ADC) and 2 (5%) of the patients had a large cell carcinoma (LCC). All patients received a total dose ≥ 60 Gy in 2 Gy-fractions. Concurrent chemotherapy was given currently with two cycles in 38 (97%) of all patients.

The majority of patients received a concurrent chemotherapy with either cisplatin/pemetrexed (36% of all patients) or cisplatin and oral vinorelbine (Navelbine) (33%) given in two cycles according to the GILT study (Flentje et al. 2016).

PD-L1 expression (median: 45%, range 1–100%) on tumor as evaluated with VENTANA PD-L1 (SP263) Assay (Roche Diagnostics, F. Hoffmann-La Roche Ltd., Basel, Switzerland) was done in 30 (77%) patients.

In the concurrent/sequential immune checkpoint inhibition group, patients receiving nivolumab were treated with a median 14 cycles (minimum 2).

Patients receiving maintenance treatment with durvalumab 10 mg/kg every bi-weekly for up to 24 cycles (median cycles 17, minimum 2 cycles).

The median follow-up of the entire cohort, SIM-I cohort, and SEQ-I cohort was 27.6, 33.3, and 26.5 months. Median survival (OS) were not achieved for the whole cohort and median PFS, PFS at 12 and 24 months were 26.3 months, 68 and 53%, respectively.

**Fig. 2:**
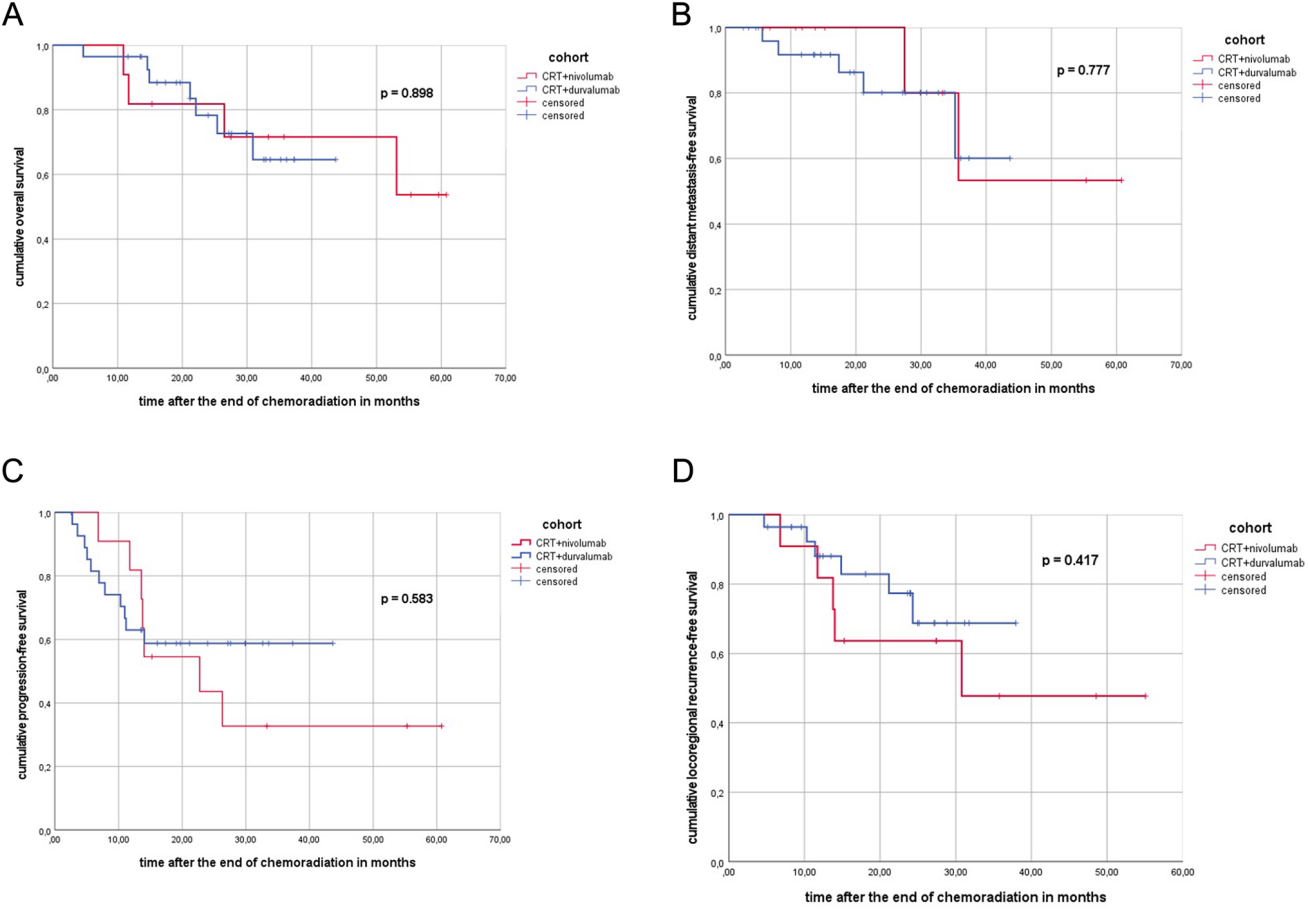
**A** Kaplan–Meier curve of the sequential/concurrent cohort (CRT + nivolumab) versus the sequential cohort (CRT + durvalumab) regarding overall survival after the end of chemoradiation in months. **B** Kaplan–Meier curve of the sequential/concurrent cohort (CRT + nivolumab) versus the sequential cohort (CRT + durvalumab) regarding distant metastasis-free survival after the end of chemoradiation in months. **C** Kaplan–Meier curve of the sequential/concurrent cohort (CRT + nivolumab) versus the sequential cohort (CRT + durvalumab) regarding progression-free survival after the end of chemoradiation in months. **D** Kaplan–Meier curve of the sequential/concurrent cohort (CRT + nivolumab) versus the sequential cohort (CRT + durvalumab) regarding locoregional recurrence-free survival after the end of chemoradiation in months

In the SIM-I cohort, the median OS and median DMFS were not reached (see Fig. 2A, B).

The median PFS and median locoregional recurrence-free survival (LRFS) was 22.8 and 30.9 months (see Fig. 2C, D).

In the SEQ-I cohort, neither median OS nor median DMFS, LRFS, BMFS or PFS could be achieved. In the SIM-I cohort, PFS at 12 and 24 months were 82 and 44% and 63 and 59% in the SEQ-I cohort (*p* = 0.583) (see Supplementary Fig. 2, Supplementary Fig. 3, Fig. 2). LRFS rate at 12 and 24 months was higher in the SEQ-I cohort with 88 and 77% versus 82 and 64% in the SIM-I cohort, (*p* = 0.417).

During follow-up, 8 (20.5%) patients suffered from IFR and 4 (10.3%) patients an OFR (4 and 2 patients each in SIM-I and SEQ-I cohort regarding IFR and OFR) (see Supplementary Fig. 2B and C).

Treatment-related side effects are shown in Table 2.

**Table 2:** Treatment-related side effects of the entire cohort and PSM-matched subgroups

Parameter	Entire cohort *N* = 39 (100%)		Nivolumab cohort *N* = 11 (28%)		Durvalumab cohort *N* = 28 (72%)	
	Any grade	Grade III	Any grade	Grade III	Any grade	Grade III
Coughing	30 (77)	0 (0)	5 (45)	0 (0)	25 (89)	0 (0)
Dermatitis	13 (33)	0 (0)	5 (45)	0 (0)	8 (29)	0 (0)
Dysgeusia	1 (4)	0 (0)	0 (0)	0 (0)	1 (4)	0 (0)
Dysphagia	22 (56)	0 (0)	6 (55)	0 (0)	16 (57)	0 (0)
Fatigue	28 (72)	0 (0)	5 (45)	0 (0)	23 (82)	0 (0)
Hoarseness	3 (8)	0 (0)	1 (9)	0 (0)	2 (7)	0 (0)
Pneumonitis	33 (85)	6 (15)	7 (64)	2 (18)	26 (93)	4 (14)
Thoracic pain	1 (4)	0 (0)	0 (0)	0 (0)	1 (4)	0 (0)
Xerostomia	1 (4)	0 (0)	0 (0)	0 (0)	1 (4)	0 (0)

Grade IV and V adverse events were not recorded. Grade III pneumonitis were found in 15% of all patients for the entire cohort. Common side effects such as radiation pneumonitis (85%), coughing (77%) and fatigue (72%) occurred in the entire cohort. In the SIM-I cohort, 18.2% of all patients developed grade III radiation pneumonitis and 14.3% in the SEQ-I cohort (*p* = 0.735). Pneumonitis CTCAE grade II was found in 36% in SIM-I and 21% in SEQ-I cohort (*p* = 0.343).

**Fig. 3:**
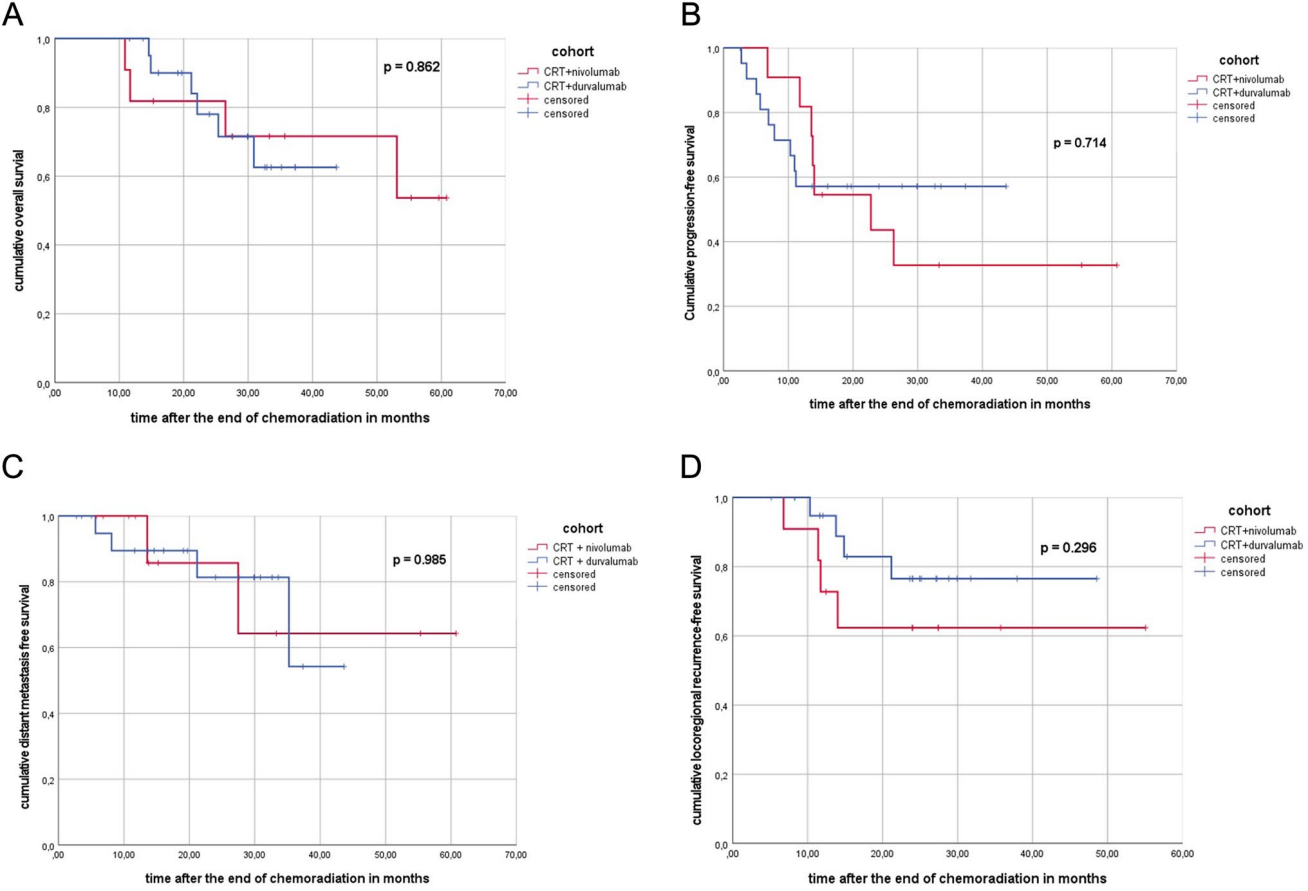

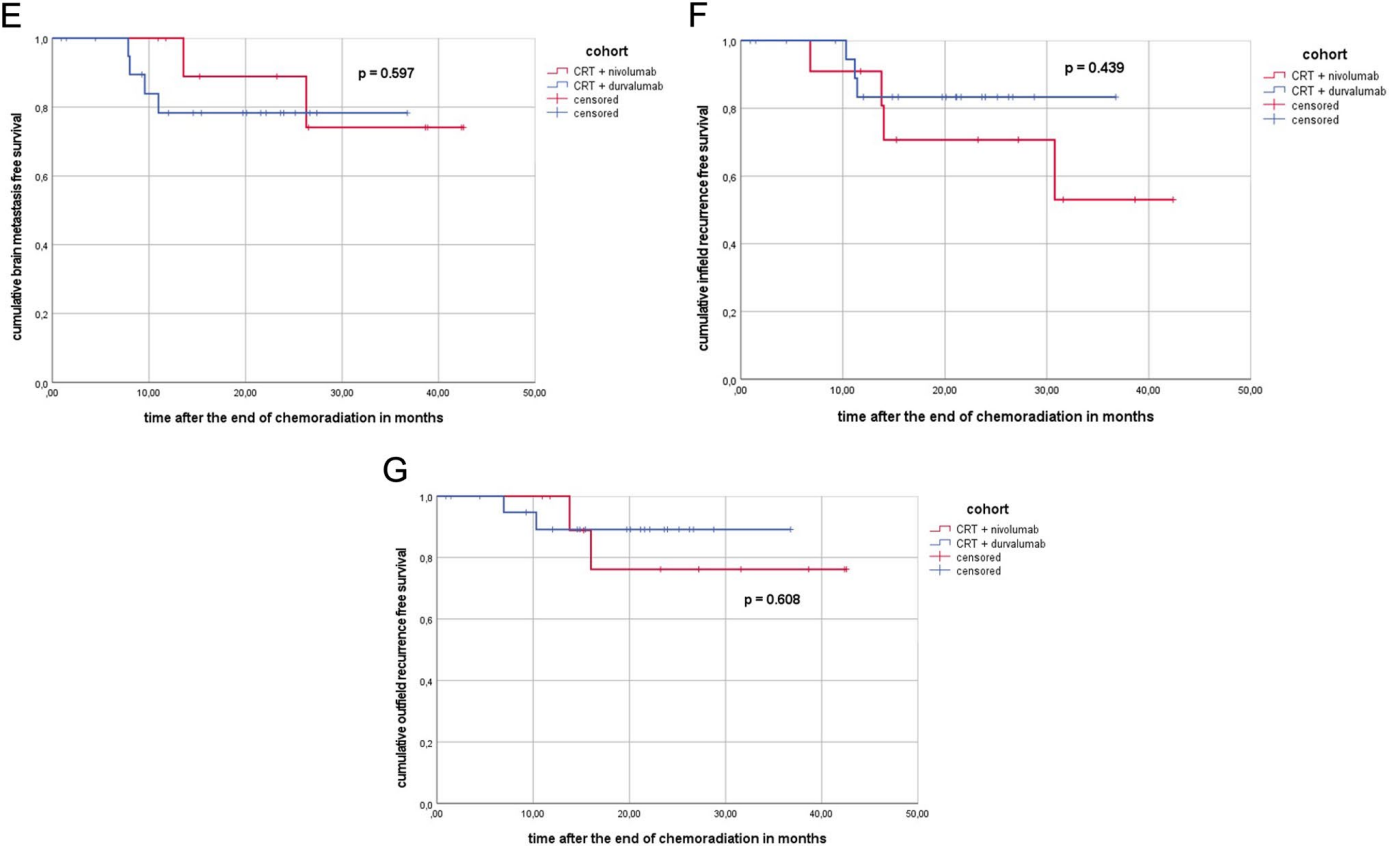
**A** Kaplan–Meier curve of the sequential/concurrent PSM cohort (CRT + nivolumab) versus the sequential PSM cohort (CRT + durvalumab) regarding overall survival after the end of chemoradiation in months. **B** Kaplan–Meier curve of the sequential/concurrent PSM cohort (CRT + nivolumab) versus the sequential PSM cohort (CRT + durvalumab) regarding progression-free survival after the end of chemoradiation in months. **C** Kaplan–Meier curve of the sequential/concurrent PSM cohort (CRT + nivolumab) versus the sequential PSM cohort (CRT + durvalumab) regarding distant metastasis-free survival after the end of chemoradiation in months. **D** Kaplan–Meier curve of the sequential/concurrent PSM cohort (CRT + nivolumab) versus the sequential PSM cohort (CRT + duivalumab) regarding locoregional recurrence-free survival after the end of chemoradiation in months. **E** Kaplan–Meier curve of the sequential/concurrent PSM cohort (CRT + nivolumab) versus the sequential PSM cohort (CRT + durvalumab) regarding brain metastasis free survival after the end of chemoradiation in months. **F** Kaplan–Meier curve of the sequential/concurrent PSM cohort (CRT + nivolumab) versus the sequential PSM cohort (CRT + durvalumab) regarding infield recurrence free survival after the end of chemoradiation in months. **G** Kaplan–Meier curve of the sequential/concurrent PSM cohort (CRT + nivolumab) versus the sequential PSM cohort (CRT + durvalumab) regarding outfield recurrence free survival after the end of chemoradiation in months

For the propensity score matching (PSM) analysis, a 1:2 ratio using age, gender, T category, tumor volume based on the planning target volume and histology was performed in order to minimize selection bias and confounding factors (*n* = 33, 11 patients in the nivolumab subgroup, 22 patients in the durvalumab subgroup). Patient and tumor characteristics of the PSM cohort are summarized in Supplementary Table 1. After PSM, PFS at 12/24 months was 82/44% in the SIM-cohort and 57/57% in the SEQ-cohort (*p* = 0.714) and LRFS at 12/24 months was 82/62 in the SIM-cohort and 95/77% in the SEQ-cohort (*p* = 0.296), respectively (see Table 3, see Fig. 3).

**Table 3:** Outcome parameter of the entire PSM cohort, PSM nivolumab and PSM durvalumab subgroup

Outcome parameter	Entire PSM cohort (%)	PSM nivolumab cohort	PSM durvalumab cohort	*p* value
Total patients	33	11	22	N/A
PFS rate at				
6 months	88	100	81	
12 months	66	82	57	
24 months	52	44	57	0.714
LRFS rate at				
6 months	100	100	100	
12 months	87	82	95	
24 months	71	62	77	0.296
IFRFS rate at				
6 months	100	100	100	
12 months	86	91	83	
24 months	79	71	83	0.439
OFRFS rate at				
6 months	100	100	100	
12 months	93	100	89	
24 months	85	76	89	0.608
DMFS rate at				
6 months	97	100	95	
12 months	93	100	90	
24 months	83	86	81	0.985
BMFS rate at				
6 months	100	100	100	
12 months	86	100	78	
24 months	87	89	78	0.597
OS rate at				
6 months	100	100	100	
12 months	94	82	100	
24 months	80	82	78	0.862

In addition, DMFS and OS at 12/24 months were 100/100 and 82/82 in the PSM nivolumab cohort and 95/90 and 100/100 in the PSM durvalumab cohort (*p* = 0.985 and *p* = 0.862) (see Fig. 3).

After PSM, the nivolumab cohort shows a grade II/III radiation pneumonitis rate of 36.4/18.2% compared to 18.2/13.6% in the durvalumab cohort (*p* = 0.258, *p* = 0.55).

## Discussion

Hereby, we report the first prospective comparative study evaluating sequential immunotherapy (standard of care) to concurrent/sequential immunotherapy with chemoradiotherapy in inoperable large stage III NSCLC using propensity score matching (PSM). Patients received the same comprehensive diagnostic and pre-treatment work-up including (PET)-CT-based treatment planning (> 96%) and brain MRI for definitive exclusion of brain metastases (> 81%).

Both treatments show a favorable side effect profile and outcomes. Concurrent/sequential immunotherapy improved early 6 and 12 months PFS, distant- and brain metastasis free survival non-significantly compared to the sequential subgroup in this small study. However, a promising and consistent 24 months PFS (57%) were found in the sequential subgroup cohort, but we have to consider that all tumors in the sequential cohort were considered PD-L1 positive (≥ 1%) compared to the concurrent/sequential subgroup (*n* = 2 PD-L1 negative, *n* = 6 unknown in the cohort). In addition, the concurrent/sequential immunotherapy subgroup could be linked to a non-significant increase of grade II and III pneumonitis, but patients in this subgroup had more advanced stages (N3 category: 23 versus 82% in PSM-SEQ versus PSM-SIM cohort, *p* = 0.001).

Our findings of concurrent/sequential immunotherapy compared to sequential immunotherapy to chemoradiation will be verified in the ongoing randomized phase III studies with PD-L1 inhibition (durvalumab: NCT04092283, NCT03519971) and PD-1 inhibition (nivolumab: NCT04026412 and pembrolizumab: NCT04380636).

To begin with, the first study investigating nivolumab given concurrently to cCRT or sequential CRT (sCRT) was the NICOLAS trial (Peters et al. 2021). After an amendment due to the promising results of the PACIFIC trial and proven feasibility of sequential nivolumab, nivolumab could be given concurrently and sequential up to by 12 months by investigators choice. Importantly, 79 patients with N category 2 or 3 in good performance status (ECOG: 0–1) before chemoradiation were analyzed and the primary endpoint of this study was early pulmonary toxicity (grade ≥ III pneumonitis up to 6 months after CRT). The median PFS was 12.7 months with a 12-months PFS of 53.7% until a median follow-up of 21 months.

According to the NICOLAS trial, there is a significant survival difference between stage IIIA and IIIB with 2-year survival rates of 81% and 56% similar to the standard arm of the PACIFIC trial (55.6%) (Peters et al. 2021; Antonia et al. 2018).

Severe pulmonary toxicity (grade ≥ III pneumonitis) was reported in only nine cases (11.7%, eight patients with grade III and one with grade V pneumonitis), all associated with nivolumab. Furthermore, one patient with grade V toxicity (esophageal ulcer with hemorrhage) was potentially related to radiotherapy and the combination with nivolumab.

Another trial investigating concurrent immunotherapy to CRT was the DETERRED trial (Lin et al. 2020). The study was divided in two parts: firstly, 10 patients received CRT with concurrent platinum-based chemotherapy. After three weeks, consolidation treatment continued with carboplatin AUC 6, paclitaxel 200 mg/m^2^ and 1200 mg atezolizumab every 3 weeks for two cycles followed by atezolizumab consolidation treatment for up to 12 months. 80% of all patients in this subgroup experienced at least one grade III or higher adverse events. In the second part, 30 patients received CRT identical to part I, followed by the same maintenance treatment as in part I. The median follow-up time and PFS were 22.5 and 18.6 months (part I) and 15.1 and 13.2 months (part II). 24 (80%) patients developed at least one grade 3 or higher CTCAE in the second part of the trial. Importantly, six (20%) patients had grade 3 or higher immune-related side effects. One (3%) patient developed a grade III pneumonitis.

In addition, the non-randomized phase II KEYNOTE-799 investigated pembrolizumab with cCRT in inoperable stage III NSCLC. After a phase I, non-randomized trial evaluating 21 patients from Jabbour et al. which confirmed safety and showed promising PFS (Jabbour et al. 2020), the study enrolled additional 216 patients from 52 participating centers in 10 countries. Primary endpoint of Keynote-799 was pulmonary toxicity (grade ≥ 3 pneumonitis) and objective response rate (ORR).

In this study, the entire patient cohort was divided into subgroups: cohort 1 consisted of 112 patients who received carboplatin AUC 6 and paclitaxel 200 mg/m^2^ every 3 weeks. After that, patients were given carboplatin AUC 2 and paclitaxel 45 mg/m^2^, weekly for 6 weeks and 2 cycles of pembrolizumab 200 mg every 3 weeks simultaneous with thoracic radiotherapy. On the other hand. cohort 2 counted 102 patients with non-squamous histology which received 3 cycles of cisplatin 75 mg/m^2^, pemetrexed 500 mg/m^2^ and pembrolizumab 200 mg every 3 weeks and thoracic irradiation during cycles 2 and 3. Importantly, both groups received consolidation treatment with pembrolizumab 200 mg every 3 weeks up to 12 months unless progression or unacceptable toxicity (grade ≥ III).

Median PFS, OS and ORR were could not be achieved in the subgroups. The 12-month PFS were 67.1 and 71.6% in cohort 1 and 2, respectively. Regarding pulmonary toxicity, grade ≥ III pneumonitis was found in 16 patients (8% in cohort 1 versus 6.9% in cohort 2).

The high number of enrolled patients is definitely a strength of this phase II study. Furthermore, the follow-up imaging and rigorous radiotherapy constraints (mean lung dose (MLD), total lung V5 and V20) is contributing to the evidence of the multimodal treatment including ICI and CRT in stage III NSCLC. However, several limitations need to be considered such as the study design, the relatively short median follow-up, exclusion of patients with Eastern Cooperative Oncology Group (ECOG) performance status 2 and the lack of reported tumor characteristics (e.g. gross tumor volume).

In summary, the NICOLAS, DETERRED and Keynote-799 trials confirmed that the concurrent application of immunotherapy with cCRT (Peters et al. 2021; Lin et al. 2020) was safe and feasible. Importantly, regarding the secondary endpoints, especially PFS, the studies resulted only in a moderate improvement, however, the achieved 12-month PFS rates was significantly improved compared to historical reports of CRT alone.

We report on the first real-world study investigating concurrent/sequential versus sequential immunotherapy in patients treated with CRT for large inoperable stage III NSCLC using the PSM method. Concurrent/sequential immunotherapy resulted to an improvement of 6 and 12 months PFS (100 versus 81%, 82 versus 57%) without statistical significance. The improvement of 12-month PFS due to concurrent ICI was also reported in Keynote-799 for both subgroups. However, we found a favorable outcome of the sequential subgroup (durvalumab) with a 24-month PFS rate of 57%. Similar, the KEYNOTE 799 trial found an estimated 2-year PFS of squamous and non-squamous NSCLC of 55.3%. In contrast to a 24-month PFS rate of our PSM-SIM cohort is in close accordance with the PACIFIC trial showing a 2-year PFS of 45% (Antonia et al. 2018; Reck et al. 2022).

Interestingly, we observed a stabilized plateau between 12 and 24 months regarding PFS in the PSM-SEQ cohort in contrast to the PSM-SIM group, whose figure steadily declined. Our findings could be explained by the advanced stage, especially the N3 category, and the lower rates of PD-L1 positive tumors in the PSM-SIM compared to the PSM-SEQ cohort.

Besides, we found a higher number of grade III radiation pneumonitis in the concurrent/sequential subgroup compared to sequential treatment (18.2 versus 13.6%, *p* = 0.55). In the PACIFIC trial, ≥ III pneumonitis was observed in 4.2% in the durvalumab cohort compared to 11.7% in the NICOLAS trial (Peters et al. 2021; Antonia et al. 2017). However, multiple Asian real-world studies reported higher rates of grade II to III pneumonitis ranging from 13 to 15% (Taugner et al. 2021; Chu et al. 2020; Jung et al. 2020). Interestingly, a meta-analysis investigating durvalumab consolidation after chemoradiation found that western studies reported less all-grade pneumonitis rates than Asian studies and elderly patients (median age > 65 years) had a higher number of all-grade pneumonitis rates than younger patients (median age ≤ 65) (Wang et al. 2021).

Several limitations need to be considered for interpreting the results. All patients were prospectively enrolled at one high volume single center but the number of patients is still quite small. Evaluating both treatments, we used PSM analysis in order to minimize selection bias and confounding variables. In addition, N category and UICC stage was significantly different in both arms while matching for age, gender, performance status, T category, planning target volume (PTV) and histology which needs to be considered carefully. Thus, durvalumab consolidation was performed based on the EMA approval and as a result, all tumors needed to have at least 1% PD-L1 expressions. However, PD-L1 expressions was unknown in 7 patients (entire cohort) due to insufficient biopsy materials. Apart of sequence, we compared PD-1 versus PD-L1 inhibition which may result in a significant bias. Based on meta-analysis data, PD-1 combinations seem superior compared to anti-PD-L1 combinations in NSCLC with conflicting data regarding toxicity, but mostly similar side effects (Brito et al. 2021; Pillai et al. 2018). In addition, one patient received sequential chemoradiotherapy in the durvalumab cohort which may impact the analyses. However, despite these limitations, our study represents the first study comparing simultaneous and maintenance treatment versus consolidation immunotherapy to chemoradiation in inoperable stage III NSCLC strongly contributing to the existing literature.

## Conclusion

Immune checkpoint inhibition either concurrent followed by consolidation or consolidation treatment alone shows a favorable toxicity profile and outcome in patients with inoperable large stage III NSCLC. Concurrent immunotherapy showed a numerical non-significant improvement regarding 6- and 12-months PFS and distant control compared to sequential approach in this small study. After PSM, we found that concurrent immunotherapy to CRT resulted in a non-significant moderate increase in grade II/III pneumonitis.

## Supplementary Information

Below is the link to the electronic supplementary material.

Supplementary file1 (DOC 30 KB)

Supplementary file2 (DOCX 68 KB)

Supplementary file3 (DOCX 75 KB)

Supplementary file4 (DOCX 18 KB)

## Data Availability

We confirm we understand the terms of the share upon reasonable request data policy, and we will make the data freely available upon request.
