# Supplementary material for: Concurrent/sequential versus sequential immune checkpoint inhibition in inoperable large stage III non-small cell lung cancer patients treated with chemoradiotherapy: a prospective observational study
Source: J Cancer Res Clin Oncol. 2023 Mar 20;149(10):7393–403. doi: 10.1007/s00432-023-04654-w (PMC10374706; doi:10.1007/s00432-023-04654-w)
Supplement: Supplementary file 1 — Supplementary file1 (DOC 30 KB) [file 432_2023_4654_MOESM1_ESM.doc]

**Supplementary Figure 1: CONSORT 2010 Flow Diagram**

**Analysis**

**Propensity score**

**matching analysis**

**(1:2 design)**

**Enrollment**

Assessed for eligibility (n= 73)

Excluded (n= 34)

  Not meeting inclusion criteria (n= 34)

Concurrent immune checkpoint inhibition

(n= 11)

Concurrent immune checkpoint inhibition

(n= 11)

Sequential immune checkpoint inhibition

(n= 22)

Sequential immune checkpoint inhibition

(n= 28)

Eligible for evaluation (n= 39)
