# Supplementary material for: Concurrent/sequential versus sequential immune checkpoint inhibition in inoperable large stage III non-small cell lung cancer patients treated with chemoradiotherapy: a prospective observational study
Source: J Cancer Res Clin Oncol. 2023 Mar 20;149(10):7393–403. doi: 10.1007/s00432-023-04654-w (PMC10374706; doi:10.1007/s00432-023-04654-w)

**Supplementary Figure IIA:** Kaplan-Meier curve of the sequential/concurrent cohort (CRT+nivolumab) versus the sequential cohort (CRT+durvalumab) regarding brain metastasis free survival (BMFS) after the end of chemoradiation in months


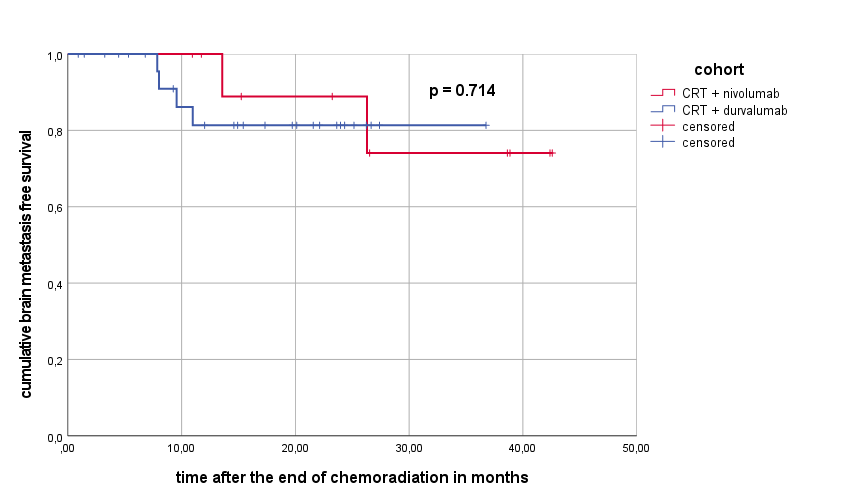


**Supplementary Figure IIB:** Kaplan-Meier curve of the sequential/concurrent cohort (CRT+nivolumab) versus the sequential cohort (CRT+durvalumab) regarding infield recurrence free survival after the end of chemoradiation in months


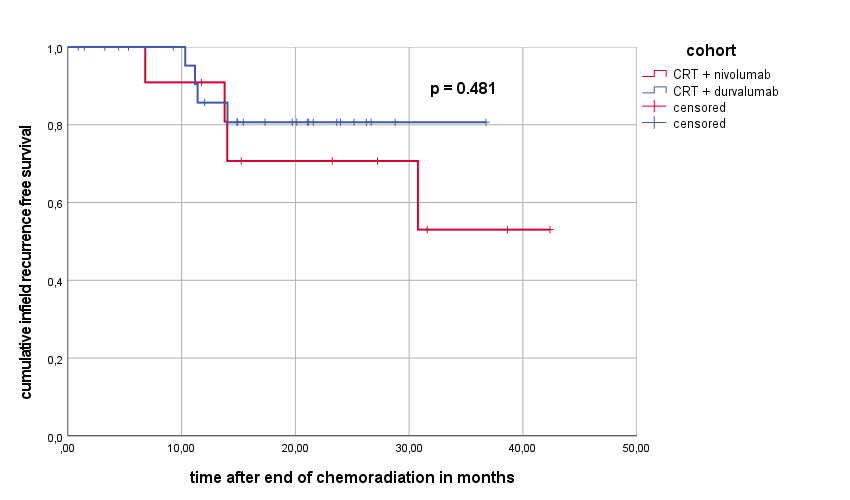


**Supplementary Figure IIC:** Kaplan-Meier curve of the sequential/concurrent cohort (CRT+nivolumab) versus the sequential cohort (CRT+durvalumab) regarding outfield recurrence free survival after the end of chemoradiation in months


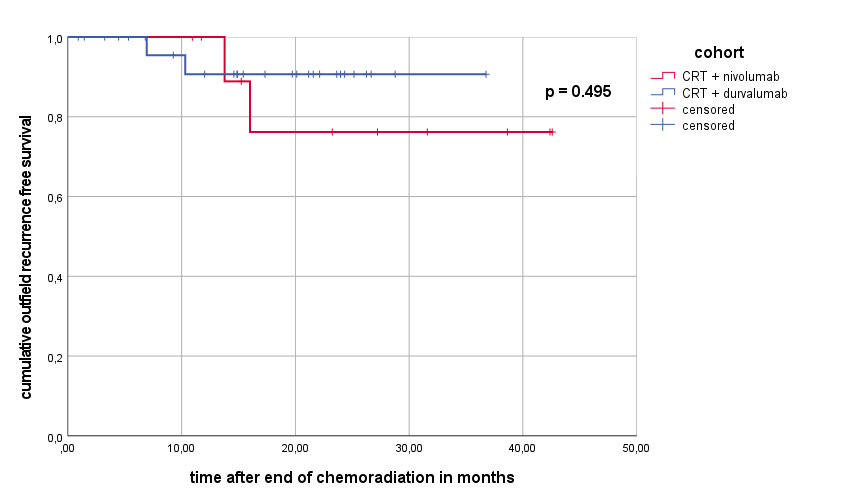

Supplement: Supplementary file 2 — Supplementary file2 (DOCX 68 KB) [file 432_2023_4654_MOESM2_ESM.docx]
