# Supplementary material for: Concurrent/sequential versus sequential immune checkpoint inhibition in inoperable large stage III non-small cell lung cancer patients treated with chemoradiotherapy: a prospective observational study
Source: J Cancer Res Clin Oncol. 2023 Mar 20;149(10):7393–403. doi: 10.1007/s00432-023-04654-w (PMC10374706; doi:10.1007/s00432-023-04654-w)

**Supplementary Figure IIIA:** Kaplan-Meier curve of the entire cohort regarding overall survival after the end of chemoradiation in months


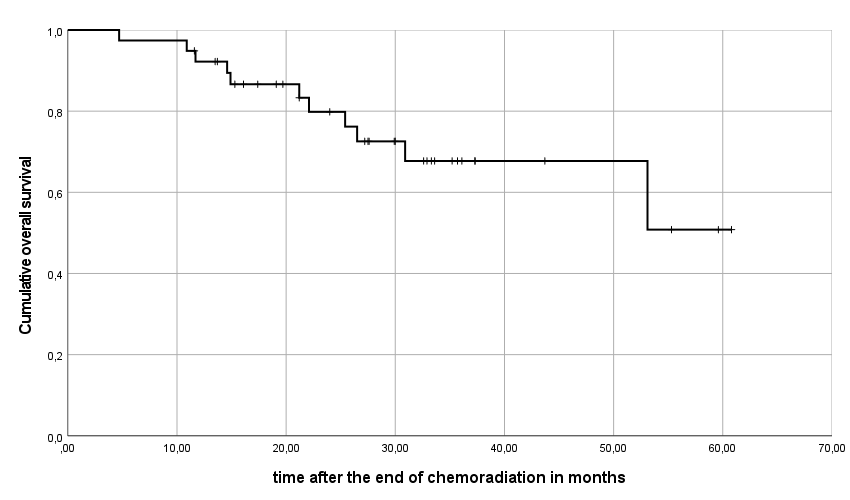


**Supplementary Figure IIIB:** Kaplan-Meier curve of the entire cohort regarding progression-free survival after the end of chemoradiation in months


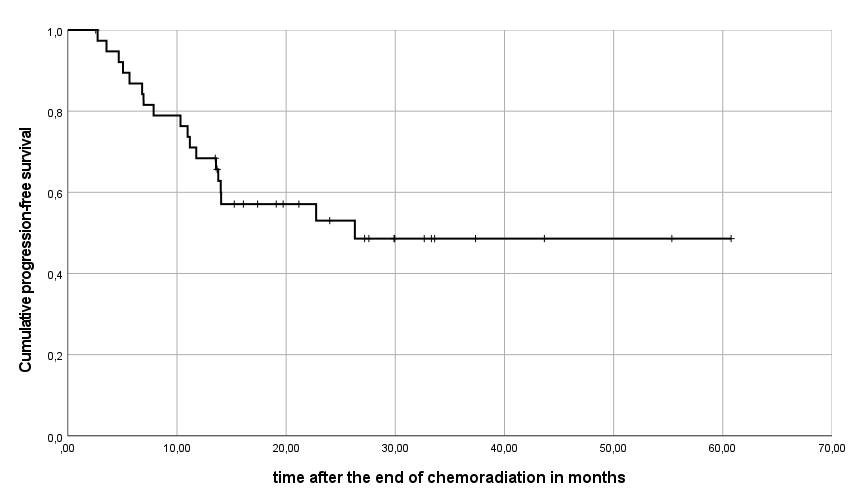


**Supplementary Figure IIIC:** Kaplan-Meier curve of the entire cohort regarding distant metastasis-free survival after the end of chemoradiation in months


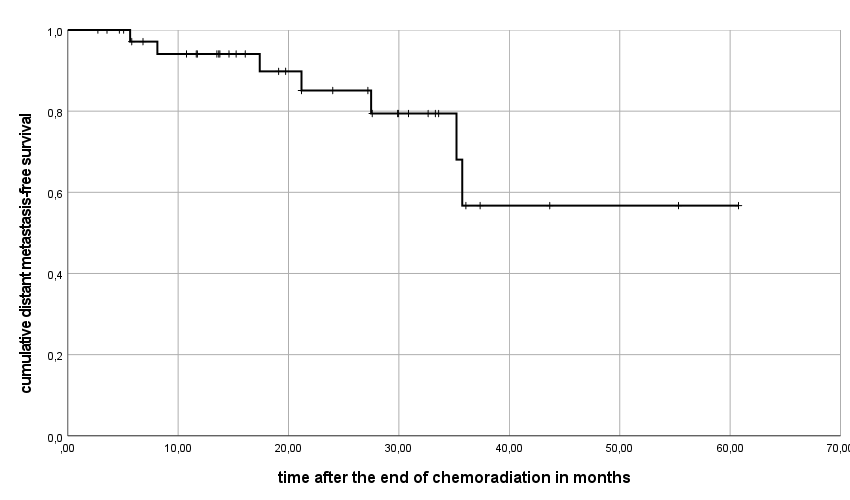


**Supplementary Figure IIID:** Kaplan-Meier curve of the entire cohort regarding locoregional recurrence-free survival after the end of chemoradiation in months


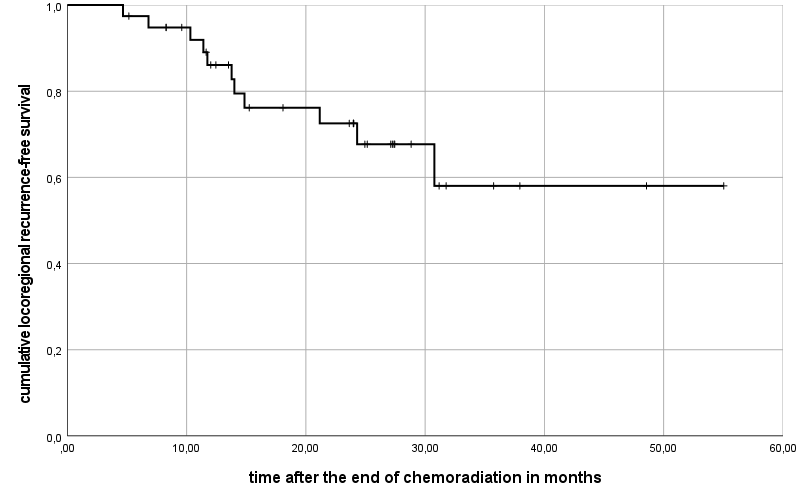

Supplement: Supplementary file 3 — Supplementary file3 (DOCX 75 KB) [file 432_2023_4654_MOESM3_ESM.docx]
