# Supplementary material for: Concurrent/sequential versus sequential immune checkpoint inhibition in inoperable large stage III non-small cell lung cancer patients treated with chemoradiotherapy: a prospective observational study
Source: J Cancer Res Clin Oncol. 2023 Mar 20;149(10):7393–403. doi: 10.1007/s00432-023-04654-w (PMC10374706; doi:10.1007/s00432-023-04654-w)
Supplement: Supplementary file 4 — Supplementary file4 (DOCX 18 KB) [file 432_2023_4654_MOESM4_ESM.docx]

| **Parameter** | **PSM**  **CRT cohort  N (%)** |  | **PSM**  **nivolumab**  **cohort** | **PSM**  **durvalumab**  **cohort** | **p-value** |
| --- | --- | --- | --- | --- | --- |
| **Total** | 33 |  | **11** | **22** |  |
| **Age, years**  Median (range) | 62 (44-77) |  | 59 (45-77) | 65 (44-72) | 0.268 |
| **Gender**  Male  Female | 25 (76) 8 (24) |  | 8 (73)  3 (27) | 17 (77)  5 (23) | 0.777 |
| **Eastern Co-operative Oncology Group performance status (ECOG PS)**  **0**  **1**  **2** | 21 (64)  11 (33)  1 (3) |  | 7 (64)  4 (36)  0 (0) | 14 (64  7 (32)  1 (5) | 0.955 |
| **T-Stage**  1  2  3  4 | 3 (9) 5 (15)  10 (30) 15 (46) |  | 2 (18) 2 (18)  1 (9) 6 (55) | 1 (5)  3 (14)  9 (41)  9 (41) | 0.951 |
| **N-Stage**  0  1  2  3 | 6 (18) 2 (6) 11 (33) 14 (42) |  | 0 (0) 0 (0)  2 (18) 9 (82) | 6 (27)  2 (9)  9 (41)  5 (23) | **0.001** |
| **UICC 8**  IIIA  IIIB IIIC | 10 (30) 14 (42) 9 (27) |  | 0 (0)  6 (55)  5 (46) | 10 (46)  8 (36)  4 (18) | **0.019** |
| **Histology**  Squamous cell carcinoma (SCC)  Adenocarcinoma (ADC)  Large cell carcinoma (LCC) | 12 (36) 19 (58) 2 (5) |  | 4 (36)  7 (64)  0 (0) | 8 (36)  12 (55)  2 (9) | 0.759 |
| **Planning target volume**  **(PTV)**  <700 ccm  ≥700 ccm  <900 ccm  ≥900 ccm | 21 (64)  12 (36)  32 (82)  7 (18) |  | 7 (64)  4 (36)  10 (91)  1 (9) | 14 (64)  8 (36)  18 (82)  4 (18) | 0.99  0.499 |
| **V20, %**  mean, SD | 24.0 (4.6) |  | 26.2 (3.9) | 22.9 (4.6) | 0.058 |
| **MLD, Gy**  mean, SD | 13.8 (2.3) |  | 15.0 (2.0) | 13.2 (2.3) | **0.026** |

Supplementary Table I. Patient characteristics of the PSM CRT cohort, PSM nivolumab and PSM durvalumab cohort
